# Thirty-year clinical outcomes after haematopoietic stem cell transplantation in neuronopathic Gaucher disease

**DOI:** 10.1186/s13023-022-02378-7

**Published:** 2022-06-18

**Authors:** Aimee Donald, Cecilia Kämpe Björkvall, Ashok Vellodi, GAUCHERITE Consortium, Timothy M. Cox, Derralyn Hughes, Simon A. Jones, Robert Wynn, Maciej Machaczka

**Affiliations:** 1 Manchester Centre for Genomic Medicine, St Marys Hospital, Manchester, UK; 2 Department of Medicine, Sunderby Regional Hospital of Norrbotten County, Luleå, Sweden; 3 Royal Manchester Children’s Hospital, Manchester, UK; 4 Great Ormond Street Hospital, London, UK; 5 GAUCHERITE Consortium, Cambridge, UK; 6 Department of Medicine, University of Cambridge, Cambridge, UK; 7 Lysosomal Storage Disorder Unit, Royal Free Hospital, UCL, London, UK; 8 Department of Human Pathophysiology, Institute of Medical Sciences, University of Rzeszow, Rzeszow, Poland; 9 Division of Internal Medicine, Department of Clinical Science and Education, Södersjukhuset, Karolinska Institutet, Stockholm, Sweden

**Keywords:** Neuronopathic Gaucher disease, Type 3 Gaucher disease, HSCT, BMT, Neurology, Outcomes

## Abstract

**Background:**

Neuronopathic Gaucher Disease (nGD) describes the condition of a subgroup of patients with the Lysosomal Storage Disorder (LSD), Gaucher disease with involvement of the central nervous system (CNS) which results from inherited deficiency of β-glucosylceramidase. Although systemic manifestations of disease are now corrected by augmentation with macrophage-targeted therapeutic enzyme (enzyme replacement therapy, ERT), neurological disease progresses unpredictably as a result of failure of therapeutic enzyme to cross the blood–brain barrier (BBB). Without therapy, the systemic and neurological effects of the disease progress and shorten life: investigators, principally in Sweden and the UK, pioneered bone marrow transplantation (BMT; Haematopoietic Stem Cell Transplantation HSCT) to supply healthy marrow-derived macrophages and other cells, to correct the peripheral disease. Here we report the first long-term follow-up (over 20 years in all cases) of nine patients in the UK and Sweden who underwent HSCT in the 1970s and 1980s. This retrospective, multicentre observational study was undertaken to determine whether there are neurological features of Gaucher disease that can be corrected by HSCT and the extent to which deterioration continues after the procedure. Since intravenous administration of ERT is approved for patients with the neuronopathic disease and ameliorates many of the important systemic manifestations but fails to correct the neurological features, we also consider the current therapeutic positioning of HSCT in this disorder.

**Results:**

In the nine patients here reported, neurological disease continued to progress after transplantation, manifesting as seizures, cerebellar disease and abnormalities of tone and reflexes.

**Conclusions:**

Although neurological disease progressed in this cohort of patients, there may be a future role for HSCT in the treatment of nGD. The procedure has the unique advantage of providing a life-long source of normally functioning macrophages in the bone marrow, and possibly other sites, after a single administration. HSCT moreover, clearly ameliorates systemic disease and this may be advantageous—especially where sustained provision of high-cost ERT cannot be guaranteed. Given the remaining unmet needs of patients with neuronopathic Gaucher disease and the greatly improved safety profile of the transplant procedure, HSCT could be considered to provide permanent correction of systemic disease, including bone disease not ameliorated by ERT, when combined with emerging therapies directed at the neurological manifestations of disease; this could include ex-vivo gene therapy approaches.

## Background

Gaucher disease (GD) is a lysosomal storage disorder (LSD) caused by pathogenic variants in the *GBA* gene (OMIM: 60646) that reduce activity of the lysosomal enzyme acid β-Glucosylceramidase. The disease is inherited as an autosomal recessive trait and classified by phenotype with traditional categorisation that depends on the presence or absence of neurological signs, most specifically the presence of slow horizontal saccades. Types ‘2’ and ‘3’ are regarded as neuronopathic (nGD); “acute” and “chronic” respectively. Infants die before childhood in the acute form of the disease and those with the chronic form, have a slowly progressive neurological disease characterised by cerebellar dysfunction, motor impairment and in some, development of seizures and myoclonus; a recent consensus definition of nGD offers clarification [1]. The systemic aspects of the disease affect all patients but until recently were considered to be the sole manifestations of ‘type 1’ disease—in which patients present with hepatosplenomegaly, a predominant thrombocytopenia (although pancytopenia occurs), bone disease and infiltration of other organs by pathological macrophages. More recently, the appearance of Parkinson disease [2] and Lewy-body dementia in a minority of patients previously classified as having type 1, non-neuronopathic Gaucher disease, has confounded the categorical clinical sub-typing.

Gaucher disease is one of more than 80 LSDs but is a paradigm for understanding disease mechanisms as well as the development of molecular and cellular therapy in the current era: it was *one* of the earliest to be characterised clinically and, from the aspect of biochemical genetics and introduction of disease-modifying treatment, is in the vanguard of contemporary therapeutics. Catalytic deficiency of β-Glucosylceramidase causes accumulation of glycosphingolipid substrates (glucosylceramide and glucosylsphingosine) in the lysosomal compartment of many cells but the molecular processes that lead to tissue injury and cell death are not understood. Many of the systemic features affect organs rich in tissue macrophages which acquire excess glucosylceramide from the engulfment and incomplete lysosomal breakdown of sphingolipids derived exogenously from other cells, especially leukocytes; the consequential enlarged pathological ‘storage’ macrophages are known as Gaucher cells and are prominent in the bone marrow, liver, and spleen. However, neuropathological studies have failed to show *consistent* accumulation of abundant Gaucher cells or overt substrate in all affected cases. It is notable that neuronal cells have the greatest glycosphingolipid content and as a result of highly disabling mutations in the *GBA1* gene, marked deficiency of β-Glucosylceramidase will limit the recycling of endogenous glycosphingolipids and probably explains the association with progressive neurological disease. Additional mechanisms of disease are likely to be implicated in the neuropathogenesis. An association between *GBA1* variants and Parkinson’s Disease (PD) has been recognised and GD-related PD and nGD are probably distinct but related neuropathological processes, the further investigation of which will aid better understanding of each.

Gaucher disease is currently treated with enzyme replacement therapy (ERT), a peripheral intravenous fortnightly infusion of recombinant enzyme which, for an nGD patient in the UK prescribed the recommended dose of 60 IU/Kg could cost around £231,000 per year (where the *NHS* (National Health Service—UK) indicative price of Cerezyme is £1071.29 and assuming a patient weight of 60 kg) [3]. This treatment has revolutionised care for patients and its efficacy in treating systemic disease has been shown repeatedly [4]. Haematological parameters normalise within months of starting treatment, growth improves, fatigue and bone events are reduced and the hepatosplenomegaly resolves. Unfortunately, ERT fails to cross the blood–brain barrier and the neurological aspects of disease in those with nGD progress uncontrolled [5, 6]. Therapies with small, potentially brain-penetrant molecules, acting as chaperones to rescue misfolded protein (resulting from missense variants) or as inhibitors of glycosphingolipid biosynthesis (substrate reduction therapy (SRT) to rebalance impaired degradation and formation of glucosylceramide) may modify neurological disease [7] in the future. At present, although Miglustat (an SRT) has been shown to cross the blood brain barrier [8] it was not successful in clinical trial in nGD [9], newer substrate reduction therapies for nGD remain in trial [10]. Patients with nGD currently have the greatest unmet needs (within the disease area) and all possibilities to explore effective therapies need consideration.

Before ERT became available, the only disease-modifying interventions were splenectomy, to relieve transfusion dependence and, in some cases, allogeneic bone marrow transplantation (BMT; Haematopoietic Stem Cell Transplantation (HSCT)). In HSCT, conditioning therapy removes host HSC and supresses immunity in the recipient so that donor HSC can engraft and are not rejected. These donors typically have normal enzyme activity (although some are *GBA1* heterozygotes) and donor HSC-derived mature progeny possessing normal β-Glucosylceramidase are widely distributed and have the potential to reconstitute deficient cellular recycling of the pathological glycosphingolipids in the brain.

In type 1 Gaucher disease, Glucosylceramidase activity in circulating leukocytes if normalised after transplant, Gaucher cells are no longer detectable and manifestations of this peripheral disease resolve. The use of allogenic BMT for Gaucher disease was replaced by ERT, in developed countries in the 1990s, because of the high procedure-related complications of transplantation. For those with neuronopathic forms of disease, in whom ERT is ineffective at controlling disease manifestations and in whom HSCT might be expected to alter the disease course, long-term outcomes have not been reported in detail.

Here we review the long term (> 20 years) of follow-up after transplantation for nGD focussing on the previously unreported neurological outcomes. Sweden and the UK share a homogeneous genotypic and phenotypic population of patients with nGD, traditionally referred to as ‘Norrbottnian’ as a result of the initial reported Swedish cohort of patients from Northern Sweden [11]. It was here that HSCT for GD was first established [12]. Most patients transplanted with Gaucher Disease originate from these two countries and here we report their long-term outcomes collectively. Our main purpose has been to determine whether neurological disease continues to progress after transplantation; we also sought to identify any confounding clinical factors, such as splenectomy [13], which may contribute to progression of the neurological features of Gaucher disease. We undertook this evaluation within this Norrbottnian cohort as the most genetically and phenotypically homogeneous cohort of patients with nGD described, all of whom harbour a common missense mutant allele, p.(Leu483Pro) (L444P), of the *GBA* gene [14]. Patients with Gaucher disease outside Sweden who carry the p.(Leu483Pro) (L444P) homozygous *GBA* genotype, show strikingly diverse clinical manifestations and severity.

Finally, we consider the cost implications of HSCT during an era in which transplant techniques have evolved, and transplant-related mortality and morbidity have greatly decreased. These aspects are considered specifically with inclusion of rare patients who failed to respond to ERT and those who have very disabling neurological manifestations or severe *GBA* variants diagnosed in early infancy. We also consider the increasingly pressing matter of the role of HSCT where the costs of ongoing enzyme therapy, or the practical capacity locally to deliver this protein intravenously, prohibit its sustained used as a first-line treatment for Gaucher disease.

## Methods

Patients who had undergone HSCT for Gaucher disease with a diagnosis of nGD in the UK and Sweden were identified by the local clinical teams (and named authors). Case records and recent clinical findings were collected. Data from the UK Gaucherite study (Predictive measures to stratify clinical outcomes in children and adults with Gaucher disease and responses to specific therapies; 2013–2018) were used to compare the outcomes of patients treated by ERT as opposed to those who were recipients of HSCT. To facilitate this comparison, the modified Severity Scoring Tool (mSST) was applied to patients [15]. This clinical scoring tool was developed for use in nGD and scores patients’ clinical features and examination findings of a total of 36 points; the higher the score, the more severely affected a patient is neurologically.

*GBA* variants are described using the former but most widespread nomenclature (where amino acids are numbered with exclusion of the first 39 amino acids of the cognate polypeptide sequence) given the familiarity of the expert community in the disease area with this terminology; however, current variant guidelines are also used here with the recommended nomenclature offered in parentheses. Contemporary nomenclature ascribes the A of the first ATG translation initiation codon as nucleotide + 1 [16].

## Results

Nine patients with a diagnosis of nGD underwent HSCT as their primary treatment between 1980 and 1990 were identified by the two national reporting groups. The clinical and demographic features are set out in Table 1.

**Table 1:**
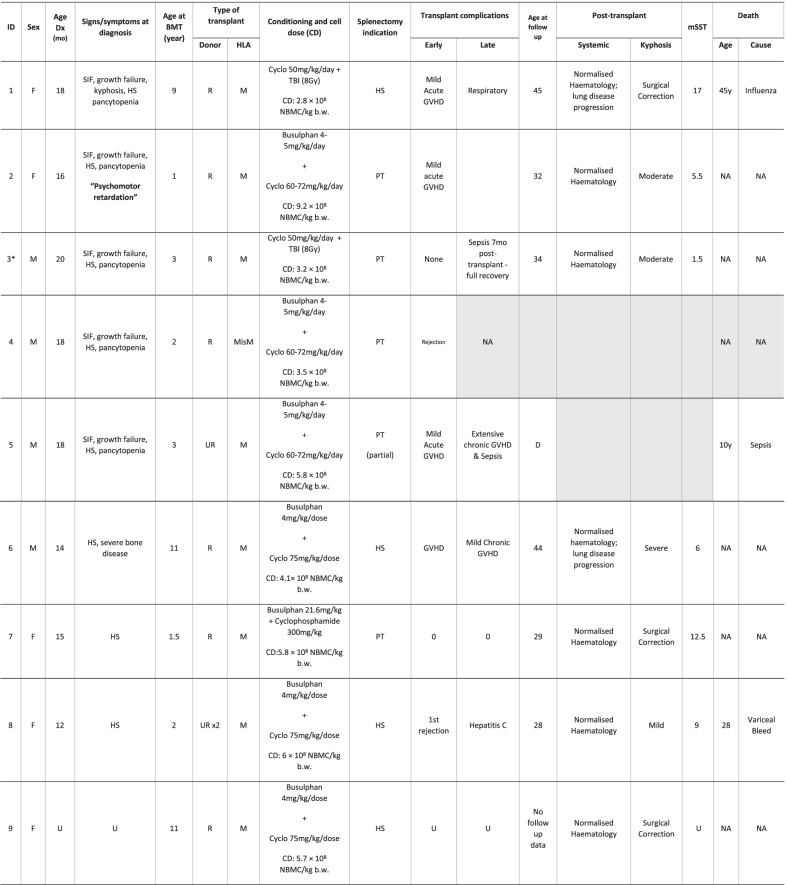
Clinical and demographic features of patient cohort

U, unknown; SIF, saccade initiation failure; HS, hepatosplenomegaly; R, related; UR, unrelated; M, matched; MisM, mismatched; CD, cell dose; NBMC, nucleated bone marrow cells; b.w., body weight; HS, hypersplenism; PT, pre-transplant; NA, not applicable; mSST, modified severity scoring too; D, died

*Genotype: A341T/L444P

Grey cells - No follow up data available due to transplant outcome

All patients shared the L444P/L444P (p.Leu483Pro) genotype, except patient 3. Age at diagnosis of Gaucher disease was 12–20 months; median 16 months; all these patients had undergone splenectomy before HSCT at age 1–9 years (median 2 years); four patients were splenectomised for hypersplenism, and five had undergone elective pre-transplant splenectomy [17]; this was a common approach in HSCT in the 1980s, with the aim of reducing the haematological recovery time after HSCT and early post-transplant mortality risks.

The transplants were from related donors in seven of nine patients; eight patients were described as HLA-matched. Of the related donors three were *GBA1* mutation heterozygotes. Age at transplant was 1–11 years (median age 3 years); cell dose received ranged from 2.8 to 9.2 × 10^8^ NBMC/kg/b.w; see Table 1. Early complications were limited to mild acute GVHD in five patients; two of these developed chronic GVHD and one rejected their graft, and this patient was later treated with ERT. A further patient rejected the first transplant and proceeded to a successful second procedure the following year (age 3 years). Persistent unwanted outcomes occurred in a further three patients; one with persistent respiratory disease attributed to the transplant (infiltration of the lungs by Gaucher cells has yet to be excluded); one who suffered septic complications as a consequence of immunosuppression but recovered fully, and one patient who acquired hepatitis C infection from a subsequent blood transfusion and died from a variceal haemorrhage at 28 years of age. At the time of reporting, two further patients had died: one from sepsis, seven years after transplantation and one from respiratory failure during acute influenza thirty-five years after the procedure. None of the patients who engrafted successfully have since needed to start ERT.

As previously reported, the biochemical outcomes [12, 18], shortly after engraftment, showed the activity of acid ß-glucosidase in peripheral blood leukocytes to briefly exceed the healthy reference range but declined to the wild type or heterozygote values. The decline was most apparent in those recipients who had less than 100% donor chimerism. More detailed enzyme and biomarker data was available for six patients and showed sustained peripheral enzyme activity at 11–21 years post-transplant and maintained chitotriosidase (clinical disease biomarker reflecting substrate burden in contemporary use for disease monitoring) within the normal range for healthy individuals (see Table 2) at up to thirty years post-transplant.

**Table 2:** Post-transplant enzyme activity and biomarker results in 6  patients

Patient		Follow up results (years’ post-transplant)	Follow up results (years’ post-transplant)
6	Enzyme activity	55% of healthy control (0.5 year)	3.10 nmol/h/mg (20 year)
	Chitotriosidase	–	54 umol/L/h (20 year)
7	Enzyme activity	7.9 nmol/h/mg (6 year)	3.6 nmol/h/mg (21 year)
	Chitotriosidase	42 umol/L/h (6 year)	47 umol/L/h (21 year)
8	Enzyme activity	10.2 nmol/h/mg (10 year)	7.6 nmol/h/mg (18 years)
	Chitotriosidase	67 nmol/h/ml (10 year)	73 nmol/h/ml (18 years)
9	Enzyme activity	4.9 nmol/mg/h (10 year)	–
	Chitotriosidase	91 umol/L.h (10 year)	–
1	Enzyme activity	–	–
	Chitotriosidase	–	11 nkat/L (30 years)
2	Enzyme activity	–	–
	Chitotriosidase	–	14 nkat/L (25 years)

Enzyme activity all measured in peripheral leukocytes—assays varied slightly by laboratory and era but all national reference validated centres

*NR Enzyme activity: 5.4–16.8; Chitotriosidase NR: < 150 nmol/h/mg or < 40 nkat/L

Pre-transplant clinical data were of limited availability. The three oldest patients at age of transplant (age 10–11 year) had documented clinical features of systemic Gaucher disease infiltration before transplantation—these included lung, liver and bone disease and presence of kyphosis [19]. No haematological parameters were available from the immediate pre-transplant period. After the procedure, haemoglobin, and platelet count at follow up age of 29–45 years was in the healthy reference range for age and gender for six patients in whom data were available. Systemic disease features at follow up included liver disease in one patient (hepatitis C with cirrhosis); bone disease in five of six patients in whom skeletal findings were noted. While predominantly, skeletal findings were spinal, one patient had clear evidence of osteonecrosis although underwent transplant at age 11 years and thus the timing of this event is unclear. Progressive kyphosis was seen in seven of seven patients, and lung disease was evident in two of six patients with data reported.

Only limited neurological findings present before transplantation were available in the records: in five patients the characteristic horizontal saccade abnormality was detected pre-transplant but was identified after the procedure in the remainder; one patient had developed abnormal vertical saccades at follow-up. No patients had any documented report of seizures, myoclonus or cerebellar signs or symptoms (ataxia, tremor, dysarthria, swallow impairment) *pre*-transplant although one patient was described as having ‘psychomotor retardation’ (patient 2). Neurological evaluation at follow-up revealed evidence of increased muscle tone in three patients, increased tendon reflexes in six patients (three also with clonus), seizures in three patients; two poorly controlled and one controlled with anticonvulsants; one additional patient described myoclonus but no seizures. Several patients had cerebellar manifestations; swallow was impaired in two and was sufficiently severe to require dietary modification; intention tremor and ataxia each occurred in three patients, and dysarthria in two. Two patients had progressive impairment of hearing, but one patient was already deaf at the time of transplant. Four patients had evidence of sleep-disordered breathing or snoring (see Table 3: Neurological Examination Post-transplant). Five patients had undergone assessment using the modified Severity Scoring Tool (mSST) with follow-up scores ranging from 1.5 to 17 (see Table 1: Clinical and Demographic Features of Patient Cohort).

**Table 3:**
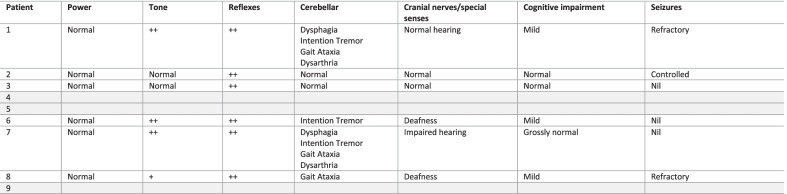
Neurological examination post-transplant

+, present; ++, increased; Grey, unknown; empty cells, not reported

All have abnormal horizontal saccades

**Fig. 1:**
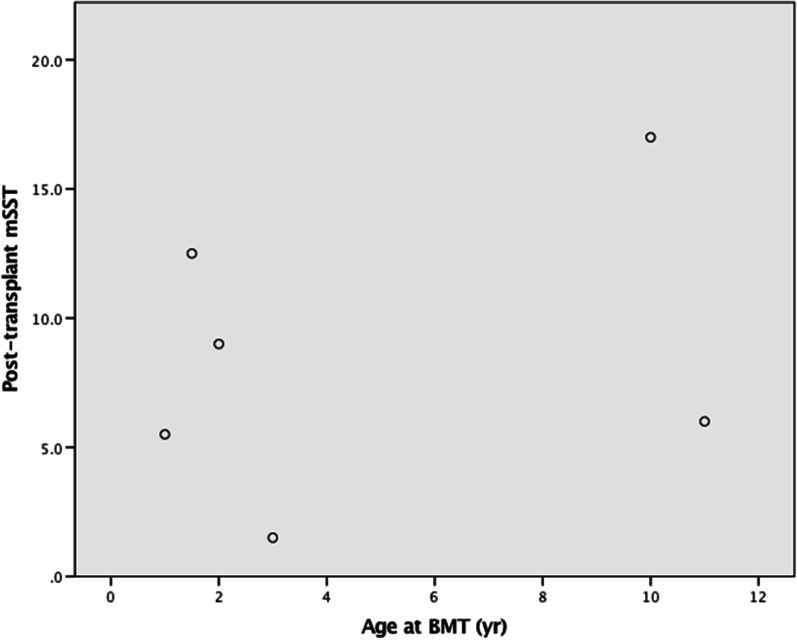
Modified Severity Score (mSST) by age at transplant; X-axis: Age at BMT in years; Y-axis: Post-transplant mSST

**Fig. 2:**
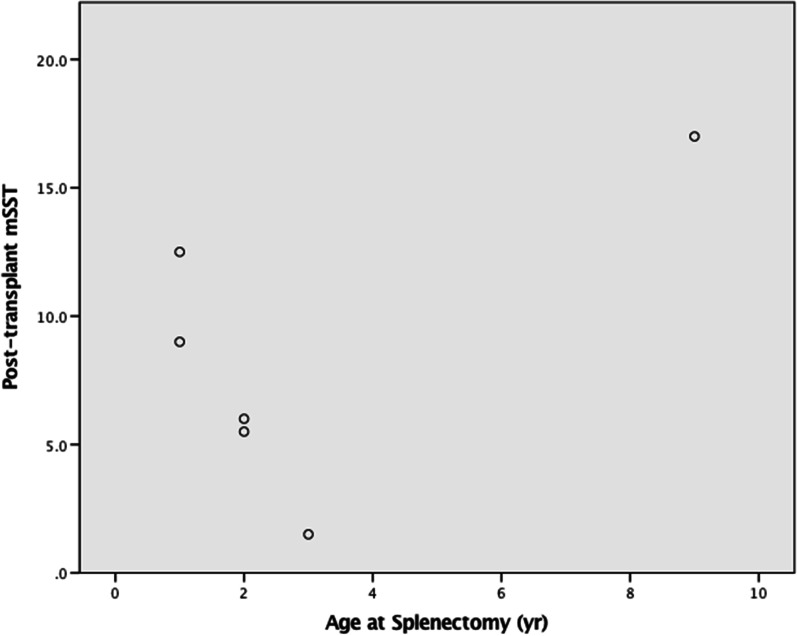
Age at Splenectomy vs mSST at follow-up; X-axis Age at splenectomy in years, Y-axis: Post-transplant mSST

Most recent mSST (follow up mSST) scores were used as a measure of disease severity: when correlated with age at transplant, no significant association was seen (see Fig. 1), although the patient with the highest mSST score was one of the oldest at the age of transplant but equally was the oldest patient in the cohort. Similarly, there was no consistent correlation between age at splenectomy and mSST score: the highest mSST score was again seen in the patient who underwent late splenectomy (age 9 years) however the next highest mSST was seen in a patient who underwent splenectomy at one year of age (see Fig. 2).

All patients received similar pre-transplantation conditioning regimes; the two who received total body irradiation (TBI) were two of the first patients to undergo transplant; they had markedly different long-term outcomes (one with an mSST of 1.5 and the other with an mSST of 17) however their pre-transplant status and timing of transplant was different; the patient with greater mSST score post-transplant was transplanted later.

Typically, patients with higher mSST scores also had a greater burden of systemic disease, but again this observation was not consistent. Those with greater systemic disease burden at follow-up tended to have greater disease burden before transplant and were often those who had undergone the procedure later but in this small cohort, the apparent trends were not statistically robust.

**Fig. 3:**
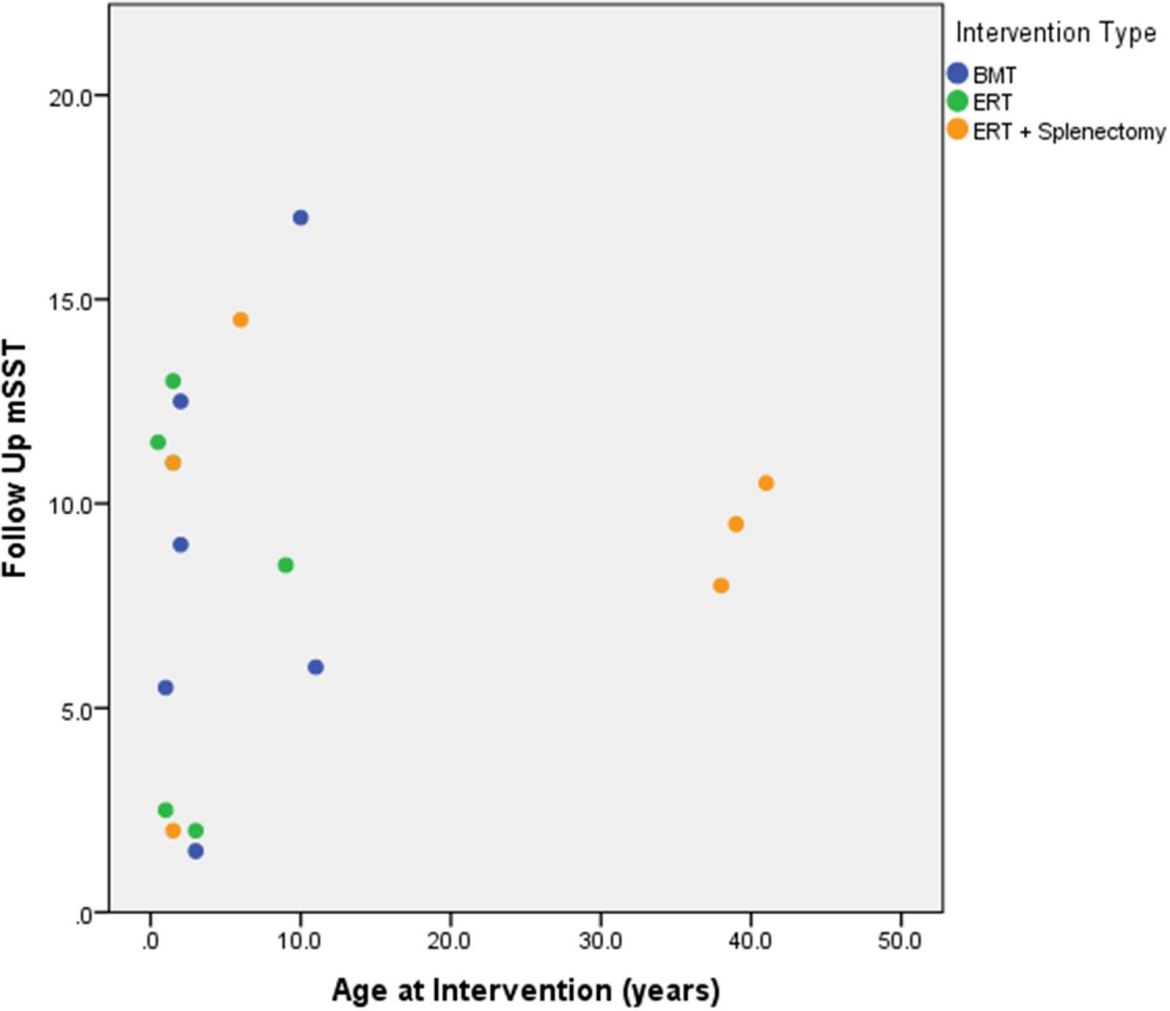
ERT vs HSCT mSST outcomes by age of intervention; X-axis Age at intervention (years); Y-axis: Follow-up mSST; Legend by Intervention type: Blue marker—Bone Marrow Transplant; Green Marker—Enzyme Replacement Therapy; Orange marker: Enzyme Replacement Therapy and splenectomy

To determine the contribution of the intervention to the outcomes, data from a cohort of patients sharing the common genotype (L444P/L444P (p.(Leu483Pro)), over the age of 20 years at follow-up, who received ERT rather than HSCT were obtained from the UK GAUCHERITE study. Most recent mSST, as an outcome measure, was compared in patients who had received a HSCT and those who received ERT and in whom the spleen had also been removed, see Fig. 3. There was no clear difference between the groups with respect to disease severity by age at intervention or by the type of intervention.

## Discussion

Review of this dataset shows that there are several salutary long-term outcomes of haematopoietic stem-cell transplantation in patients with neuronopathic Gaucher disease. It is a notable feature that sustained benefit of intervention was seen in the context of a non-malignant disease, even though the procedure was carried out early in the era of its introduction as a clinical therapy. A further point is that when, as is desirable, the donors were related, graft failure was very unusual.

Haematopoietic stem-cell transplantation—initially using stem cells harvested from bone marrow donors—was pioneered in the 1960s but generally successful outcomes using HLA-matched donors for acute lymphoid as well as myeloid leukaemia’s were reported after 1977 by the Seattle group led by E. Donnall Thomas [20]. These outcomes were reported only six years in advance of the first transplant in the cohort of Gaucher patients reported here, reflecting the ambitious engagement of investigators in the UK and Sweden (led by John Hobbs and Olle Ringdén, respectively). Of the nGD patients described, all survived the transplant procedure for at least five years. Deaths attributable to the effects of the procedure occurred in two patients but were unrelated to HSCT and attributable to the manifestations of Gaucher disease, in the third and oldest recipient.

Chronic graft-versus-host disease affected many patients in this cohort and in this sense, the outcome in patients with Gaucher disease in no exception [21]. In a few patients, the contribution of GVHD to reduced quality of life can be difficult to distinguish from the neuronopathic or somatic effects of Gaucher disease in the recipient, but in any clinical situation this complication is clearly a strong determinant of health outcomes from the procedure. There have however been several advances in allogeneic HSCT in recent years resulting in a significant improvement in long term post-transplant outcomes, including lower mortality and a lower incidence of chronic GVHD.

Efficacy measures may be conveniently considered with respect to the systemic effects of Gaucher disease in peripheral tissues and those that reflect the central manifestations of the disease in the nervous system. All the patients in the cohort had correction of haematological parameters with restoration of acid β-glucosidase activity in circulating leucocytes that indicates correction of marrow disease after engraftment by donor stem-cells. Apart from the patient with cirrhosis due to hepatitis C, liver disease was not found in the HSCT recipients. Lung infiltrates have been noted in patients with nGD and two patients in this cohort have developed lung infiltration although detailed information about pulmonary function or high-resolution radiological imaging were not available and thus could not be firmly distinguished from chronic pulmonary GVHD. Given the extent and clinical importance of skeletal disease in nGD, none of the patients developed new osseous lesions or symptomatic skeletal disease (defined by episodes of osteonecrosis, bone crises or fragility fractures) however most had evidence of mild bone disease.

Kyphosis and scoliosis are distinctive musculoskeletal complications of neuronopathic Gaucher disease. All patients in the cohort showed progression of this deformity after HSCT.

While the lack of good objective measures of neurological disease burden is a limitation in all areas of clinical research in this disease, it is clear from this study that despite HSCT, the neurological features in this group of patients continue to progress slowly. The mSST gives a comparable estimation of CNS impairment although this is limited by the subjectivity of the examiner and their interpretation of the performance of the patient. Onset of seizures is however an unmistakable, and single, clinical feature that often provides a helpful level of objectivity but, as in this series, is not universal. When compared with patients who received ERT rather than HSCT, our impression was of a slower rate of neurological progression in the HSCT cohort; while this has been noted previously [12], we concede that the absence of repeated systematic mSST scoring over a long period of follow-up in all patients detracts from the strength of this claim. In the past, the neurological burden in Gaucher disease and other lysosomal diseases has been determined by measures of intellectual function, most often Wechsler Intelligence Scales. Unfortunately, these scales were not carried out in this nGD cohort. Furthermore, used in this way, the value of Wechsler Intelligence Scales is unclear—especially given the strong evidence of preserved intellectual function in many patients with nGD and the lack of overt or consistent changes in cognitive function over time [22]. It is possible that the neurological progression observed in our cohort was slower than might have been expected had they not received HSCT. However, in the absence of any good control data it is not possible to definitively say this.

In this cohort, splenectomy had been carried out for debilitating hypersplenism in three patients, the remainder underwent splenectomy as a pre-transplant procedure. There are risks and benefits to splenectomy in HSCT and appraisal of the attendant risks have changed with experience of transplantations. In the setting of hypersplenism accompanied by marked pancytopenia, while expedient or necessary, splenectomy was originally considered to worsen the substrate burden on the bone marrow and CNS, worsening disease [23]. Splenectomy also had sepsis risks [17] which in the current era are better understood and complications prevented with use of prophylactic immunisation and antibiotic and antifungal regimes. It was recognised that pre-transplant splenectomy in those without hypersplenism resulted in slower engraftment but a significantly lower requirement for transfusion [23]. There have been several successful transplants in patients with Gaucher disease without splenectomy and it is the opinion of the authors that the transfusion requirements in transplant with modern conditioning regimes is lower overall. However, the health of the spleen prior to transplant should be assessed and a decision on the need for splenectomy made on an individual basis. A contemporary approach might be to utilise pre-transplant ERT for a defined time period, with a view to reducing splenomegaly and risks of hypersplenism.

The challenge of treating neurological features of nGD can be best resolved by consideration of the blood brain barrier (BBB). This protective system maintains homeostasis by preventing rapid fluctuations in the cerebral environment which would be generated if peripheral molecules, many of which act as central neurotransmitters, could freely enter. Large molecules which are not lipid-soluble therefore are prevented from crossing the barrier of tight junctions between capillary endothelial cells [24]. Since the mannose-terminated human β-glucosylceramidase infused intravenously in Gaucher disease (as ERT) is a large glycoprotein of molecular size ≈ 70 KDa and unable to traverse the blood–brain barrier, it is widely accepted that enzyme therapy does not affect the natural progression of the neurological features. Similar junctions have the same effect at the blood-CSF- barrier which lies between the choroid plexus and the CSF. Exploitation of specific transporter mechanisms is one method of transferring therapeutics to the CSF, however a series of ATP-Binding Cassette (ABC) transporters help to further maintain the CSF environment by removing neurotoxins actively and therefore even if passage of the BBB is achieved, retention in the CNS is the next challenge.

Enzyme therapy targets the macrophage via mannose receptors; consideration of the neuropathology underlying nGD is essential when generating new therapeutic approaches. Microglia are resident macrophages of the CNS and thus the presumed cellular target in the neurological disease. Although microglial activation has been demonstrated in Gaucher disease [25], Wong et al. [26] showed that neurons and astroglia are the primary cells implicated in neuropathology. Neuronal loss and astrogliosis may be a direct consequence of Gaucher cell formation within the perivascular spaces, which could trigger a local neuroinflammatory response. Recent advances in understanding the immune regulation in the CNS has identified another population of macrophage cells, Perivascular Macrophages (PVMs) [27] which could be implicated in Gaucher disease. The location and function of these cells would make them susceptible to transformation into pathological Gaucher cells. That HSCT would be expected to ameliorate the neuropathological effects by replacing these resident immune cells in the nervous system by peripheral donor macrophages has been used to justify transplantation in other LSDs characterised by neurodegeneration. However, PVMs and microglial populations are not primarily recruited from peripheral blood monocyte precursors but are derived from haematopoietic cells in the embryonic period and then maintained by a process of self-renewal [27]. A population of ‘bone-marrow derived myelomonocytes’ does circulate, and in mouse studies, has indeed been shown to be capable of repopulating the brain [28]. Where the blood–brain barrier is disrupted, it seems probably that this process will be more efficient and this can be utilised as a means to complement deficient lysosomal enzyme activity. However, in the case of neuronopathic Gaucher disease, the capacity of healthy donor cells to complement glucosylceramidase activity by cross-correction is exceptionally limited and cell-autonomous corrective effects will be dominant. In any event, the choice of conditioning regime is likely to be critical for ensuring optimal therapeutic efficacy. Busulfan is the conditioning treatment of choice in HSCT for its myeloablative properties and contemporary regimens have tolerable toxicity; Busulfan treatment also decreases macrophage populations in the brain and disrupts the BBB in a manner that appears to enhance central donor engraftment, although functional reconstitution is a gradual process [29]. Mouse studies have shown that while irradiation can induce effective CNS engraftment in disorders such as Rett syndrome, due to greater and more rapid disruption of the BBB, this occurs at the expense of greater long-term toxicity [30]. In this context, it is noteworthy that one of the two patients in this cohort of Gaucher patients who received Total Body Irradiation (TBI), as part of their conditioning for HSCT, has the lowest follow-up mSST score, perhaps indicating the benefit of improved engraftment. However, the other patient who received TBI had a much higher mSST score at follow up and so radiation toxicity could equally be implicated in the poorer outcome. However, in this particular case, progressive disease seems more likely to reflect the interplay of confounding factors; they were older age at the time of transplant and had a greater burden of pre-transplant disease. We contend, that conditioning protocols remain important determinants of neurological outcome and regimens that employ Busulfan are safer and better tolerated than irradiation. Finally, it seems clear from many studies that protocols based on Busulfan achieve greater central engraftment (of donor microglia) in the context of disease affecting the brain [31]. However, it is also clear that the measures used to determine functional outcomes are equally important and should relate specifically to the disease of interest. Disease-specific measures are critical in order to differentiate the neurotoxicity of the conditioning protocol from the neuropathological changes attributable to the disease in the recipient. In patients who undergo HSCT for haematological cancers, a neurocognitive decline is typically more severe and re-acquisition of skills after the transplant is worse in those who had received conditioning with irradiation [32].

The aforementioned features: age at transplant and pre-transplant disease status, have been implicated as determinants of outcome in other lysosomal storage disorders which utilise HSCT as a primary therapeutic strategy. In mucopolysaccharidoses I (Hurler syndrome) long term outcome studies have shown younger age at transplant and lower neurological disease burden pre-transplant, were predictive for better neurodevelopmental outcome, while conditioning with TBI was a predictor of inferior outcome [33].

Perhaps the most successful outcomes of HSCT for a neurodegenerative, metabolic disease, occur in X-linked Adrenoleukodystrophy (X-ALD). This disorder results from inherited defects of an integral membrane protein of the peroxisome rather than an enzyme deficiency, the resultant tissue and plasma accumulation of very long chain fatty acids (VLCFAs) causes progressive demyelination. HSCT was used in this disease before the underlying genetic and biochemical defect was identified but has been shown to attenuate and reverse the demyelination with striking outcome, particularly when carried out before symptoms develop. Although the basis of this effect is not yet fully explained, the question, as for some other diseases, including lysosomal diseases, is raised as to whether correction of central enzyme deficiency can relieve the neurological disease by a systemic improvement in the disposal of toxic metabolites. In X-ALD at least, is has been suggested that the immune system (specifically microglial) function in the nervous system is defective, and that repopulation of the microglia with donor-derived macrophages, the CNS immune response to accumulation of VLCFA subsequent neuroinflammation is suppressed. A similar mechanism may theoretically operate in Gaucher disease, Gaucher cell formation may exhaust the capacity of perivascular macrophages and microglia and disrupt the innate immune and homeostatic system in the CNS. The impaired immune function may lead to neuronal loss and reactive astrogliosis. In these circumstances, the very restricted ability of Glucosylceramidase to participate in cell-to-cell functional complementation after HSCT may therefore be irrelevant to outcome since cell-autonomous reconstitution of immune function will be required to overcome the neuroinflammatory disturbance in the nervous system due to local overproduction of toxic glycosphingolipids by neurones. In these circumstances, depletion of resident immune cells and maximisation of donor engraftment may be the greater priority. In the same manner, if a safe method of restoring recipient enzyme function in the resident neural cells is employed, for example by clinically approved ex-vivo gene therapy, this needs to occur before the immune cells are transformed into Gaucher cells, i.e., very early in the pre-symptomatic period.

In conclusion, while this cohort of patients is too small to confidently identify the predictors of outcome; the importance of age at time of transplant and pre-transplant disease burden appear consistent with experience in other LSDs. We also suggest that the conditioning regimes used should be considerate of the effect on the BBB to maximise central engraftment.

The cost–benefit analysis of HSCT for neuronopathic Gaucher disease is unclear; but the financial cost is clearly much lower for HSCT compared with long-term ERT and this is a matter that is relevant to health care systems where funding excludes ERT as an option or where for other reasons sustained life-long intravenous infusions are impractical. In a centre with specialised experience, and the use of contemporary, safer, conditioning regimens, the adverse outcomes associated with HSCT are limited and relieves patients of the burden of lifelong IV ERT.

Patients with the most severe forms of nGD (type 2) are rarely offered any attempt at a definitive therapeutic intervention; the disease progresses rapidly, and patients are too unwell to tolerate the conditioning required for HSCT. There may be circumstances however, when this policy merits further consideration.

In the current era of advanced therapies, the described experience of HSCT in this disease serves as a foundation for ex-vivo gene therapy approaches. This current report demonstrates successful engraftment using donor cells to achieve normal range, sustained, glucocerebrosidase activity and normalised biomarkers of disease activity. It therefore suggests that an ex-vivo gene therapy approach using autologous stem cells would offer the same benefits as HSCT but with a lower conditioning requirement (and therefore lower toxicity). Importantly, such an approach, with a CNS target can be manipulated using promoters and specific vectors to be integrating and therefore achieve long term enzyme production, to target specific cells e.g. neurones and to over-express enzyme with a view to achieving supraphysiological activity which has been suggested to be beneficial in other neuronopathic lysosomal disorders [34]. Given the severity of the neurological manifestations, combinatorial strategies also involving other modalities of treatment may ultimately be needed to fully rescue this disease for healthy long-term functioning in those affected.

## Conclusion

We have shown that patients with nGD who have undergone HSCT can have comparable outcomes to those patients who receive ERT. There is a wealth of contemporary experience of HSCT in other inherited neurodegenerative and neurometabolic disorders which offers an opportunity to re-evaluate the role of HSCT for nGD. Furthermore, there is an obligation to consider HSCT as a treatment option for nGD, particularly in the context of a patient for whom no other therapy is available and to use this experience to develop more innovative approaches.

## Data Availability

The datasets used in this study may be available with restrictions to some details (for patient confidentiality) from the corresponding author on reasonable request.

## References

[1] Schiffmann R, Sevigny J, Rolfs A, Davies EH, Goker-Alpan O, Abdelwahab M (2020). The definition of neuronopathic Gaucher disease. J Inherit Metab Dis.

[2] Neumann J, Bras J, Deas E, O’Sullivan SS, Parkkinen L, Lachmann RH (2009). Glucocerebrosidase mutations in clinical and pathologically proven Parkinson’s disease. Brain.

[3] Joint Formulary Committee. British National Formulary (online). London: BMJ Group and Pharmaceutical Press; n.d.

[4] Cabrera-Salazar MA, O’Rourke E, Henderson N, Wessel H, Barranger JA (2004). Correlation of surrogate markers of Gaucher disease Implications for long-term follow up of enzyme replacement therapy. Clin Chim Acta.

[5] Schiffmann R, Heyes MP, Aerts JM, Dambrosia JM, Patterson MC, DeGraba T (1997). Prospective study of neurological responses to treatment with macrophage-targeted glucocerebrosidase in patients with type 3 Gaucher’s disease. Ann Neurol.

[6] Altarescu G, Hill S, Wiggs E, Jeffries N, Kreps C, Parker CC (2001). The efficacy of enzyme replacement therapy in patients with chronic neuronopathic Gaucher’s disease. J Pediatr.

[7] Cabrera-Salazar MA, DeRiso M, Bercury SD, Li L, Lydon JT, Weber W (2012). Systemic delivery of a glucosylceramide synthase inhibitor reduces CNS substrates and increases lifespan in a mouse model of type 2 gaucher disease. PLoS ONE.

[8] Treiber A, Morand O, Clozel M (2007). The pharmacokinetics and tissue distribution of the glucosylceramide synthase inhibitor miglustat in the rat. Xenobiotica.

[9] Schiffmann R, FitzGibbon EJ, Harris C, DeVile C, Davies EH, Abel L (2008). Randomized, controlled trial of miglustat in Gaucher’s disease type 3. Ann Neurol.

[10] Marshall J, Sun Y, Bangari DS, Budman E, Park H, Nietupski JB (2016). CNS-accessible inhibitor of glucosylceramide synthase for substrate reduction therapy of neuronopathic gaucher disease. Mol Ther.

[11] Blom S, Erikson A (1983). Gaucher disease—Norrbottnian type. Eur J Pediatr.

[12] Erikson A, Groth CG, Månsson J-E, Percy A, Ringdén O, Svennerholm L (1990). Clinical and biochemical outcome of marrow transplantation for Gaucher disease of the Norrbottnian type. Acta Pædiatr.

[13] Erikson A (1986). Gaucher disease: Norrbottnian type (III)—neuropaediatric and neurobiological aspects of clinical patterns and treatment.

[14] Dahl N, Hillborg P-O, Olofsson A (1993). Gaucher disease (Norrbottnian type III): probable founders identified by genealogical and molecular studies. Hum Genet.

[15] Davies EH, Mengel E, Tylki-Szymanska A, Kleinotiene G, Reinke J, Vellodi A (2011). Four-year follow-up of chronic neuronopathic Gaucher disease in Europeans using a modified severity scoring tool. J Inherit Metab Dis.

[16] den Dunnen JT, Dalgleish R, Maglott DR, Hart RK, Greenblatt MS, McGowan-Jordan J (2016). HGVS recommendations for the description of sequence variants: 2016 update. Hum Mutat.

[17] Hobbs JR, Shaw PJ, Jones KH, Lindsay I, Hancock M (1987). Beneficial effect of pre-transplant splenectomy on displacement bone marrow transplantation for Gaucher’s syndromE. The Lancet.

[18] Ringden O, Groth CG, Erikson A, Granqvist S, Mansson JE, Sparrelid E (1995). Ten years’ experience of bone marrow transplantation for Gaucher disease. Transplantation.

[19] Ringden O, Groth C-G, Erikson A, Backman L, Granqvist S, Marnsson J-E (1988). Long-term follow-up of the first successful bone marrow transplantation in Gaucher disease. Transplantation.

[20] Henig I, Zuckerman T (2014). Hematopoietic stem cell transplantation—50 years of evolution and future perspectives. Rambam Maimonides Med J.

[21] Pavletic SZ, Fowler DH (2012). Are we making progress in GVHD prophylaxis and treatment?. Hematology.

[22] Steward AM, Wiggs E, Lindstrom T, Ukwuani S, Ryan E, Tayebi N (2019). Variation in cognitive function over time in Gaucher disease type 3. Neurology.

[23] Ringden O, Groth CG, Erikson A, Mansson JE, Svennerholm L. Bone Marrow transplantation in the Norrbottnian type of Gaucher’. Correction of Certain Genetic Diseases by Transplantation 1989;30–5.

[24] Tajes M, Ramos-Fernández E, Weng-Jiang X, Bosch-Morató M, Guivernau B, Eraso-Pichot A (2014). The blood-brain barrier: structure, function and therapeutic approaches to cross it. Mol Membr Biol.

[25] Norman RM, Urich H, Lloyd OC (1956). The neuropathology of infantile Gaucher’s disease. J Pathol.

[26] Wong K, Sidransky E, Verma A, Mixon T, Sandberg GD, Wakefield LK (2004). Neuropathology provides clues to the pathophysiology of Gaucher disease. Mol Genet Metab.

[27] Faraco G, Park L, Anrather J, Iadecola C (2017). Brain perivascular macrophages: characterization and functional roles in health and disease. J Mol Med (Berl).

[28] Vallieres L, Sawchenko PE (2003). Bone marrow-derived cells that populate the adult mouse brain preserve their hematopoietic identity. J Neurosci.

[29] Capotondo A, Milazzo R, Politi LS, Quattrini A, Palini A, Plati T (2012). Brain conditioning is instrumental for successful microglia reconstitution following hematopoietic stem cell transplantation. Proc Natl Acad Sci.

[30] Derecki NC, Cronk JC, Lu Z, Xu E, Abbott SBG, Guyenet PG (2012). Wild-type microglia arrest pathology in a mouse model of Rett syndrome. Nature.

[31] Wilkinson FL, Sergijenko A, Langford-Smith KJ, Malinowska M, Wynn RF, Bigger BW (2013). Busulfan conditioning enhances engraftment of hematopoietic donor-derived cells in the brain compared with irradiation. Mol Ther.

[32] Shah AJ, Epport K, Azen C, Killen R, Wilson K, De Clerck D (2008). Progressive declines in neurocognitive function among survivors of hematopoietic stem cell transplantation for pediatric hematologic malignancies. J Pediatr Hematol Oncol.

[33] Aldenhoven M, Wynn RF, Orchard PJ, O’Meara A, Veys P, Fischer A (2015). Long-term outcome of Hurler syndrome patients after hematopoietic cell transplantation: an international multicenter study. Blood.

[34] Eichler F, Duncan C, Musolino PL, Orchard PJ, De Oliveira S, Thrasher AJ (2017). Hematopoietic stem-cell gene therapy for cerebral adrenoleukodystrophy. N Engl J Med.

